# Leveraging Factors of Self-Efficacy and Motivation to Optimize Stroke Recovery

**DOI:** 10.3389/fneur.2022.823202

**Published:** 2022-02-24

**Authors:** Rachana Gangwani, Amelia Cain, Amy Collins, Jessica M. Cassidy

**Affiliations:** ^1^Department of Allied Health Sciences, University of North Carolina at Chapel Hill, Chapel Hill, NC, United States; ^2^Human Movement Sciences Curriculum, University of North Carolina at Chapel Hill, Chapel Hill, NC, United States

**Keywords:** stroke, self-efficacy, motivation, neurorehabilitation, neuroimaging, biomarker

## Abstract

The International Classification of Functioning, Disability and Health framework recognizes that an individual's functioning post-stroke reflects an interaction between their health condition and contextual factors encompassing personal and environmental factors. Personal factors significantly impact rehabilitation outcomes as they determine how an individual evaluates their situation and copes with their condition in daily life. A key personal factor is self-efficacy—an individual's belief in their capacity to achieve certain outcomes. Self-efficacy influences an individual's motivational state to execute behaviors necessary for achieving desired rehabilitation outcomes. Stroke rehabilitation practice and research now acknowledge self-efficacy and motivation as critical elements in post-stroke recovery, and increasing evidence highlights their contributions to motor (re)learning. Given the informative value of neuroimaging-based biomarkers in stroke, elucidating the neurological underpinnings of self-efficacy and motivation may optimize post-stroke recovery. In this review, we examine the role of self-efficacy and motivation in stroke rehabilitation and recovery, identify potential neural substrates underlying these factors from current neuroimaging literature, and discuss how leveraging these factors and their associated neural substrates has the potential to advance the field of stroke rehabilitation.

## Introduction

Stroke is a heterogeneous condition resulting in profound and wide-ranging effects on physical, psychological, and social aspects of an individual's life (1, 2). Neurorehabilitation is an important component in an individual's recovery post-stroke (3). The World Health Organization (WHO) International Classification of Functioning, Disability and Health (ICF) model is a universally recognized framework for health and disability (4) that delineates various levels of disability in stroke, including impairments, activity limitations, and participation restrictions (5). Rehabilitation therapists and clinicians utilize this framework to guide evaluation, treatment strategies, and goal-setting post-stroke (6).

Early stroke rehabilitation practice primarily focuses on recovery of impairments in the body structure and function domain of the ICF to optimize physical functioning. Such an approach is frequently based on motor learning principles of providing intensive, progressive, and task-specific interventions to improve physical function and capacity (i.e., what a person can do in a standardized, controlled environment post-stroke) (7, 8). However, improvement in physical capacity does not translate to improvement in physical performance (i.e., what a person actually does in his or her daily environment) (9). This suggests that improvement in the body structure and function domain may not necessarily translate to improvement in social participation or reintegration into pre-stroke life roles. Expanding the focus of post-stroke rehabilitation beyond the domain of body structure and function may therefore promote more meaningful outcomes that translate to real-world participation.

A relevant feature in the ICF model is the inclusion of contextual factors, including environmental and personal factors (10). The latter refers to factors that are not a part of an individual's health condition or health state. Personal factors include gender, age, co-morbidities, socioeconomic status, education, and behavioral characteristics such as self-efficacy and motivation (11). These personal factors relate to performance and participation outside of the clinical environment (12). Self-efficacy relates to “beliefs in one's capabilities to organize and execute courses of action required to produce given attainments” (13). Individuals with high self-efficacy post-stroke typically have greater confidence to participate in activities of daily living (ADLs), higher ability to overcome barriers in their recovery, and typically possess greater psychosocial functioning and well-being compared to those with low self-efficacy (14, 15). As recent work indicates baseline ADL function as an important prognostic factor of functional independence during early post-stroke recovery (16), self-efficacy also contributes to post-stroke recovery and rehabilitation outcomes. Closely related to self-efficacy is motivation, which refers to an individual's will to perform a certain behavior toward achieving their goal and is one of the processes through which self-efficacy affects human functioning and impacts post-stroke rehabilitation outcomes (17). An individual's level of self-efficacy influences their motivation as exemplified in the following ways: determination of goals that individuals set for themselves, determination of effort expended by an individual in achieving their goal(s), determination of how long an individual perseveres when faced with challenges, and determination of an individual's resilience to failure (18). Given the importance of self-efficacy and motivation in goal-setting, perseverance, and resilience, these personal factors are key contributors to recovery and rehabilitation processes, including those related to stroke.

Motor learning theories such as the Dynamic Systems Theory and OPTIMAL (Optimizing Performance through Intrinsic Motivation and Attention for Learning) Theory have incorporated personal factors to explain motor (re)learning post-stroke (19, 20), whereby the optimization of learning and recovery depend on an individual's level of self-efficacy and motivation. Interventions aimed at enhancing these personal factors have demonstrated a reduction in functional decline 3–12 months post-stroke (21), significant improvement in rehabilitation outcomes including the Reintegration to Normal Living Index and Activities-specific Balance Confidence (ABC) Scale (22), and significant functional recovery at 6 months post-stroke (23). Further, such interventions in individuals with chronic conditions, including stroke, demonstrated lasting improvement in coping strategies including symptom management, increased physical activity, less fatigue, and fewer hospital visits due to secondary complications (24).

The assessment of self-efficacy and motivation primarily entails self-report scales and questionnaires, which introduce limitations related to response bias and subjectivity with scoring (25, 26). Brain-based measures acquired through neuroimaging may provide greater objectivity. Past research examining neuroimaging-based biomarkers of stroke recovery have shown the utility of these measurements in describing post-stroke injury and behavioral status as well as predictive value in post-stroke recovery and treatment response (27–29). Expanding biomarker development to elucidate neural substrates subserving self-efficacy and motivation may hold important clinical implications. Identifying neural correlates of self-efficacy and motivation, for instance, may provide information beyond what conventional measures alone convey. This information may result in a more objective assessment of self-efficacy and motivation to better tailor motor (re)learning and treatment strategies. In this review, we identify potential neural substrates underlying motivation, self-efficacy, and constructs of self-efficacy such as self-agency from current neuroimaging literature, and discuss how leveraging these factors and their associated neural substrates has the potential to advance the field of stroke rehabilitation.

There are a number of systematic reviews on self-efficacy and motivation in stroke focusing on the role of these factors in stroke rehabilitation outcomes (30–32) and their implementation in treatment and intervention strategies (30, 33). Findings from these systematic reviews have demonstrated significant associations between self-efficacy and post-stroke outcomes such as quality of life, activities of daily living (ADLs), mobility, and depression (30, 31) and have encouraged the incorporation of these factors in rehabilitation programming (33) and medical curriculum (31). This review examines the roles of self-efficacy and motivation in stroke rehabilitation and recovery while distinguishing itself from other reviews by bridging topics of self-efficacy and motivation with neuroimaging and biomarker development.

## An Overview of Self-Efficacy and Motivation

Albert Bandura's Social Cognitive Theory describes learning as a dynamic process arising from the interaction between person, environment, and behavior to explain goal-directed behavior and its maintenance across time (34, 35). Self-efficacy is an important feature of Social Cognitive Theory that refers to the control of human action through an individual's beliefs in their capabilities to produce desired outcomes by their actions (34).

Since self-efficacy impacts stroke rehabilitation (e.g., sustaining progress and coping with setbacks), understanding how self-efficacy beliefs originate is important. Self-efficacy beliefs arise from the following instances: (1) Performance mastery. Successful performance experiences raise mastery and efficacy expectations. Once established, enhanced self-efficacy tends to generalize to other situations in which performance was lacking (36). In individuals with stroke, the enhancement of self-efficacy occurs through the accomplishment of therapy goals through independent effort. (2) Vicarious experience. Observing others perform activities generates expectations in observers that they may achieve similar outcomes with persistent effort (37). (3) Verbal persuasion. Individuals believe in their ability to successfully cope with adverse experiences based on the encouragement from others, including health care professionals and family members (38). (4) Emotional arousal. An individual's emotional state influences their level of self-efficacy (38). For example, stressful situations may perpetuate emotions that negatively affects an individual's self-efficacy or their perceived ability to accomplish rehabilitation goals.

While performance mastery, vicarious experiences, verbal persuasion, and emotional arousal shape one's degree of self-efficacy, self-efficacy impacts human functioning through several psychological processes (39). (1) Cognitive processes. Individuals' self-efficacy influences the anticipatory scenarios that they mentally construct and rehearse. In rehabilitation settings, for example, individuals with high self-efficacy, visualize successful scenarios entailing positive rehabilitation outcomes, whereas those with low self-efficacy, visualize failure scenarios. In adverse situations, those with low self-efficacy tend to lower their aspirations, which negatively impacts their performance. In contrast, those with high self-efficacy, typically establish and pursue ambitious goals for themselves (40). (2) Motivational processes. Self-efficacy is key in the self-regulation of motivation (41). Individuals motivate themselves and guide their actions based on their beliefs of what they can do and set goals for themselves and plan the course of action accordingly. Thus, high self-efficacy results in greater motivation to set ambitious yet attainable rehabilitation goals along with effective planning to execute behaviors necessary to achieve those goals. (3) Affective processes. Self-efficacy is also key in anxiety arousal. Individuals with high self-efficacy typically manage anxiety and stressful conditions in a productive manner during rehabilitation; whereas, those with low self-efficacy cannot (42). (4) Selection processes. Self-efficacy influences the types of activities that individuals decide to pursue (42). Those with diminished self-efficacy actively avoid activities during rehabilitation that they believe exceed their capabilities (42). In contrast, those with heightened self-efficacy readily undertake challenging activities and select situations that they judge themselves capable of handling. A construct of self-efficacy relevant to stroke rehabilitation discussed below is *self-agency*, which refers to the belief that one's action is the consequence of one's intention (43). Bandura's Social Cognitive Theory describes *agency* as an individual's ability to control and regulate their thinking, motivation, and behavior with existing self-beliefs (i.e., self-efficacy) (43). Motivation, self-efficacy, and constructs of self-efficacy such as self-agency therefore play a central role in post-stroke recovery and rehabilitation outcomes.

## Self-Efficacy and Motivation in Stroke Rehabilitation

Until recent years, stroke rehabilitation often emphasized impairment mitigation; however, evidence suggests that impairment mitigation does not necessarily translate to improved participation in daily activities or enhanced quality of life (9). Thus, it is necessary to consider and evaluate all aspects of the ICF model to develop a more thorough understanding of stroke recovery. Further, as rehabilitation outcomes depend on patients' attitudes, self-beliefs, and motivation, post-stroke outcomes are therefore contingent on an individual's ability to actively participate in the rehabilitation process. Yet, barriers to post-stroke recovery, including depression (44) and anxiety (45), may compromise an individual's level of self-efficacy and motivation thereby impacting physical capacity and participation (46) and resulting in lower engagement during rehabilitation (47). For instance, recent work by Stewart et al. (48) that examined self-efficacy for reach speed and accuracy in individuals with chronic stroke displaying mild motor impairment found self-efficacy to be a significant predictor of affected arm reaching performance (48). Complimenting these findings is work showing a significant positive association between individuals' level of motivation at the start of inpatient post-stroke rehabilitation and level of independence with ADLs at the end of rehabilitation as measured by the Functional Independent Measurement scale (49). In addition to engagement in the rehabilitation process, low self-efficacy and motivation may also negatively influence treatment adherence (50, 51). Work by Caetano et al. (52) revealed that self-efficacy for walking, along with walking ability, explained 80% of the variance in exercise adherence in individuals post-stroke (52). As these collective findings imply, self-efficacy and motivation influence rehabilitation outcomes, and this likely occurs through several routes. First, as a primary focus of stroke rehabilitation is to facilitate an individual's volitional movement, a patient's motivation and self-efficacy beliefs influence their motor behavior (20). High self-efficacy and motivation enhance future expectations and generate autonomy, which may translate to individuals setting ambitious rehabilitation goals and a stronger commitment to achieve those goals (20). Second, self-efficacy and motivation influence the perceived demand related to task performance, which may further impact an individual's task preparation during rehabilitation (53). Individuals with high self-efficacy and motivation thus focus more on achievable elements of the task and less on potential shortcomings. Third, enhanced expectations arising from high self-efficacy positively impact cognitive processes such as working memory and attention (54), which are imperative for effective (re)learning following stroke. Lastly, enhanced expectations are related to increased dopaminergic mediation, which modulates motivation to guide future behavior (55). Thus, self-efficacy and motivation can enhance post-stroke rehabilitation outcomes and participation by influencing various motivational, cognitive and physiological aspects to ensure that goals are effectively coupled with desired actions in real-life.

Self-efficacy and motivation are also incorporated in self-management and home-based rehabilitation programs to promote long-term behavioral change and its maintenance over time following inpatient hospitalization (56). Post-stroke self-management programs led by therapists incorporating self-efficacy and motivation factors focus on goal-setting and empowering individuals with information, support and resources to facilitate post-acute care transition and symptom management (15, 57, 58). Such self-management strategies have resulted in improved confidence in recovery, better long-term health outcomes, and a reduction in post-stroke complications (33, 59, 60) as evidenced by increased physical activity, improved self-reported mobility and fine motor-skill performance, and elevated balance confidence (61, 62). The recent Taking Charge after Stroke (TaCAS) study examined a novel self-management program in community dwelling individuals within 16 weeks post-stroke (63). As a departure from the SMART (Specific, Measurable, Achievable, Realistic/relevant, and Timed) approach, the Take Charge intervention instead incorporated elements of Self-Determination Theory (64) by fostering autonomy, confidence, and purpose. Participants explored and discussed important aspects of their lives, including people, along with priorities that they hoped to address over the next 12 months. Compared to control participants that received written stroke education, individuals completing the Take Charge intervention demonstrated significantly higher quality of life scores (Short Form 36 Physical Component Summary, SF-36) and significantly lower odds of dependency (modified Rankin scale 3–5) at 12 months post-stroke. A notable finding of the TaCAS study was a significant dose effect with higher quality of life scores observed with an additional Take Charge session (63).

Other interventions incorporating self-efficacy and motivation factors delivered by rehabilitation therapists include individualized coaching (65) and cognitive strategy training (66). Individualized coaching is a patient-centered process that aims to facilitate and empower the individual to achieve self-determined goals related to their health and wellness (67). In individuals with stroke, personalized coaching involving goal-setting, monitoring of goals, and motivation counseling resulted in improved physical activity behavior and participation as measured by the number of weekly exercise sessions, intensity and duration of exercise, and step count at 1 year post-stroke (68). Similarly, cognitive strategy training incorporates an integrated approach from behavioral and cognitive psychology fields. Here, a therapist guides the patient in goal-setting and facilitates skill acquisition through a guided recovery process where the patient (learner) identifies a problem and uses feedback and guidance from the therapist to generate potential solutions (69). Such training enhanced patients' self-monitoring capabilities and problem-solving skills that led to successful rehabilitation outcomes (70–72). Rehabilitation approaches and strategies integrating concepts of self-efficacy and motivation therefore have the potential to optimize post-stroke recovery outcomes through enhanced patient autonomy and participation during the recovery process.

## Assessment of Self-Efficacy and Motivation

Measurement of self-efficacy typically occurs through self-reports and questionnaires whereby individuals rate their degree of confidence in performing a specific task (73). One measure of self-efficacy, referred to as *self-efficacy magnitude*, is determined by summing the total positive or *yes* responses from an individual wherein a greater number of positive responses implies greater self-efficacy (73). Another measure, *self-efficacy strength*, is determined by summing the confidence ratings across all performance levels with higher scores representing greater confidence levels (74). For example, the General Self-Efficacy Scale (GSES) (75) is a 10-item psychometric scale that rates an individual's level of self-efficacy based on their self-beliefs of meeting task demands in a broad array of contexts. Responses utilize a 4-point Likert Scale with scores ranging between 10 and 40 (higher scores suggest higher self-efficacy). Similarly, several subjective patient-based measures of motivation exist (25, 76). However, these scales demonstrate limited reliability and validity with notable methodological limitations (77). The overall subjective nature of self-efficacy and motivation questionnaires therefore likely limits their widespread use in rehabilitation. Establishing greater objectivity in the assessment of self-efficacy and motivation would benefit both research and clinical settings by providing a more holistic understanding of the individual.

## Neuroimaging and Biomarker Development

The application of structural and functional neuroimaging in stroke rehabilitation propelled the development of biomarkers or measurements reflecting underlying cellular and molecular events associated with clinical status and/or evolution (27). Stroke recovery biomarkers have the potential to enhance the accuracy of post-stroke recovery and treatment response prediction (27). For instance, structural neuroimaging measures derived from magnetic resonance imaging (MRI), diffusion tensor imaging (DTI), and transcranial magnetic stimulation that convey corticospinal tract integrity have informed motor recovery and treatment outcomes in stroke (78–80). Similarly, functional neuroimaging measures reflecting cortical oscillatory activity and functional connectivity as measured by electroencephalography, magnetoencephalography, and functional MRI, respectively, have also demonstrated similar associations with post-stroke recovery of motor (81), somatosensory (82), language (83), and cognitive function (84). Collectively, these measurements of brain structure and physiological function have provided a more comprehensive understanding of stroke recovery. Additionally, evidence also suggests greater prediction accuracy with the use of neuroimaging biomarkers in conjunction with clinical outcome measures vs. clinical outcome measures alone (85). While the emphasis of these neuroimaging measures has encompassed mostly improvement and recovery of impairment and function, the utility of these measures may also apply to the assessment of personal factors such as self-efficacy and motivation. The identification of pertinent neural correlates (i.e., relevant neural structures and connections) of self-efficacy and motivation may enhance the accuracy of existing post-stroke recovery prediction models.

## Neural Correlates of Self-Efficacy

There is a limited but growing body of literature utilizing neuroimaging to identify potential neurological correlates of self-efficacy and its constructs such as self-agency (Table 1). Most studies in existence predominantly involve young adults with no significant neurological history. One of the largest studies to date conducted by Nakagawa et al. (91) involved 1,204 young adults (91). Using a combination of MRI and DTI measures, the investigators determined that higher general self-efficacy scores related to lower mean diffusivity (higher neuronal density) from the lenticular nucleus (putamen and globus pallidus). These findings compliment previous work demonstrating contributions from the putamen to motor control and skill acquisition (93, 94). Previous research findings have also shown that putamen volume positively correlates with perceptual-motor performance and that functional connections between the sensorimotor cortex and the posterior putamen strengthen in parallel with learning (95). Collectively, these findings substantiate the contributions of the putamen in both motor learning and self-efficacy processes. The findings by Nakagawa et al. (91) also align with work illustrating contributions from the globus pallidus in the development and control of learning in humans (96). The corticostriatal loop connects cortical motor planning regions with subcortical structures, including the thalamus, putamen, and globus pallidus to efficiently execute and control motor behavior (97). Together, these findings underscore the importance and relevance of the lenticular nucleus as a neural substrate of both self-efficacy and motor learning and control.

**Table 1:** Neural correlates of self-efficacy identified in healthy individuals.

**References**	**N**	**Age (years)**	**Imaging**	**Neural correlate(s)**
Farrer and Frith (86)	12	29	fMRI	AIC, R IPL
Yomogida et al. (87)	28	18–24	fMRI	SMA, L CB, R PPC, R EBA
Nahab et al. (88)	20	18–33	fMRI	PPC, STS, DLPFC, pre-SMA, precuneus, insula, CB
Davis et al. (89)	79	65–75	MRI	Total brain and gray matter volumes
Kang et al. (90)	19	22–35	EEG	Alpha (8–12 Hz) band oscillations, anterior frontal area
Nakagawa et al. (91)	1,204	20.7 ± 1.8	MRI	Lenticular nucleus
Hirao (92)	89	19.7 ± 0.6	fNIRS	L PFC

*Age presented as range or mean ± standard deviation. N, Number of study participants; AIC, anterior insular cortex; CB, cerebellum; DLPFC, dorsolateral prefrontal cortex; EBA, extrastriate body area; EEG, electroencephalography; fMRI, functional magnetic resonance imaging; IPL, inferior parietal lobule; L, left; MRI, magnetic resonance imaging; fNIRS, functional near-infrared spectroscopy; PFC, prefrontal cortex; PPC, posterior parietal cortex; R, right; SMA, supplementary motor area; STS, superior temporal sulcus*.

In addition to subcortical structures, Hirao (92) also identified the prefrontal cortex as a crucial region of self-efficacy. Using functional near-infrared spectroscopy (fNIRS), they compared changes in prefrontal activation during a verbal fluency task across 89 healthy young adults previously categorized into low, moderate, and high self-efficacy groups. Investigators found significantly less left prefrontal activation in the low self-efficacy group as compared to the moderate self-efficacy group, which infers potential involvement of prefrontal cortical activity in self-efficacy. These findings supplement past work showing contributions from prefrontal cortex in self-regulation or behaviors that result in the fulfillment of one's intended goals (98, 99). Combined, these findings suggest the importance of prefrontal cortex in both self-efficacy and self-regulation in goal-directed behavior. Future investigation is necessary to determine how these findings translate to the rehabilitation environment where outcomes depend on the consistency of goal-directed behavior. In a study in 79 community-dwelling older women, Davis et al. (89) specifically assessed self-efficacy related to falls using the ABC Scale and found that falls self-efficacy positively correlated with both total brain and gray matter volumes. These findings resonate with previous findings that found that increased risk of falls in those with advancing age occurs in part due to decreases in brain volume and reduced cognition (100). Given the occurrence of falls post-stroke and the impact of stroke on brain volume (101), determining the predictive value of total brain volume on falls self-efficacy may be an effective future research direction.

While the aforementioned studies focus on neural correlates of self-efficacy, several studies of young adults with no neurological conditions (86–88, 90) have also examined neural correlates of self-agency. As previously defined, self-agency is a construct of self-efficacy that refers to an individual's ability to influence their own functioning by regulating their thinking, motivation, and behavior with existing self-beliefs to achieve desired outcomes (43). Work employing functional MRI (fMRI), identified several cortical regions and neural structures associated with self-agency (86–88): anterior insula and the right inferior parietal lobule, posterior parietal cortex (PPC), superior temporal sulcus (STS), dorsolateral prefrontal cortex (DLPFC), pre-supplementary motor area (pre-SMA), precuneus, insula, cerebellum, supplementary motor area (SMA), right posterior parietal cortex (PPC), and right extra striate body area (EBA). Relatedly, a meta-analysis encompassing 15 fMRI studies across 228 study participants identified activation of the insula as a neural correlate of self-agency (102).

As expected, several regions associated with self-efficacy also relate to self-agency, and these regions, in turn, contribute to motor system function (ventral premotor cortex, SMA, pre-SMA, and cerebellum) and to cognition and information processing (DLPFC, PPC, and insula) (103). The anterior insula, for instance, shares connections with sub-regions of the prefrontal cortex, such as the dorsolateral and ventromedial prefrontal cortices, that control attention and working memory (104). These cognitive processes are imperative for motor relearning post-stroke and impact subsequent rehabilitation outcomes (105). Similarly, recent neurophysiological and neuroimaging evidence acknowledges the involvement of PPC and premotor regions in internal monitoring of self- and externally-generated movements (106), which are essential components in relearning and regaining movement post-stroke. Additional studies are needed to provide a definitive understanding of how neural structures concomitantly involved in self-agency and motor and cognitive processing influence post-stroke rehabilitation outcomes.

Apart from the anatomical structures associated with self-agency, EEG work involving a virtual reality motor paradigm in a healthy cohort highlighted neural oscillatory activity in the alpha (8–12 Hz) band (90). Decreases in relative alpha power, specifically overlying central, bilateral parietal, and right temporal areas, related to greater self-agency. Additionally, significant decrease in alpha coherence (connectivity) involving regions overlying anterior frontal cortex negatively correlated with self-agency. Past work demonstrating the involvement of neural oscillations in the alpha frequency band in stroke recovery (107, 108) encourage additional work to validate these findings in a stroke cohort.

## Neural Correlates of Motivation

Akin to the self-efficacy literature, most studies examining neural correlates of motivation involve fMRI in healthy adults (Table 2). Given the close relationship between self-efficacy and motivation, many of the anatomical regions previously reported above have also been identified in studies of motivation. In 161 young adults (19–24 years of age), Lee and Reeve (110) found that greater anterior insular cortex activation related to greater motivation (110), which aligns with other work highlighting greater interactions between anterior insular cortex and striatum associated with greater motivation (109, 114). Together, these findings substantiate past research showing contributions from the ventral and dorsal anterior insula to an individual's motivational state with the latter region specifically contributing to the updating of motivational states according to associated goal-directed actions (116). Other regions identified by fMRI include prefrontal, sensorimotor, middle cingulate, and visual cortices (111, 112, 115) along with nucleus accumbens and precuneous (112), with greater recruitment of these regions associated with greater motivation. Interestingly, in an effort to establish a causal link between motivation and frontopolar (FPC) cortex, Soutschek et al. (117) stimulated FPC with transcranial direct current stimulation (tDCS) in 141 healthy adults (117). Facilitation of this region with anodal tDCS increased participant motivation to put forth additional effort necessary to obtain a reward. This is consistent with prior work highlighting the FPC in goal-directed behavior (118). Determining how these findings extrapolate to neurorehabilitation is a necessary next step.

**Table 2:** Neural correlates of motivation identified in healthy individuals.

**References**	**N**	**Age (years)**	**Imaging**	**Neural correlate(s)**
Schmidt et al. (109)	20	19–27	fMRI	BG
Lee and Reeve (110)	161	19–24	fMRI	AIC
Quirin et al. (111)	17	23.6 ± 3.2	fMRI	L PFC
Radke et al. (112)	36	19–48	fMRI	NA, MCC, precuneus
Myers et al. (113)	20	11.2 ± 2.1	fMRI	Connectivity between striatum and medial PFC and dorsal/rostral ACC
Lee and Reeve (114)	22	22.9 ± 2.8	fMRI	Connectivity between AIC and striatum
Kohli et al. (115)	100	20–46	fMRI	Striatum, midbrain, sensorimotor and occipital cortices

*Age presented as range or mean ± standard deviation. N, Number of study participants; AIC, anterior insular cortex; ACC, anterior cingulate cortex; BG, basal ganglia; fMRI, functional magnetic resonance imaging; L, left; MCC, middle cingulate cortex; NA, nucleus accumbens; PFC, prefrontal cortex*.

Emerging work in pediatric neuroimaging has also identified potential key regions associated with motivation particularly related to *grit* and *growth mindset* (113). The former refers to the long-term perseverance toward a goal and the latter refers to the belief that effort improves talent. The investigators found that *grit* positively correlated with ventral striatal networks including structural connectivity to medial prefrontal and rostral anterior cingulate cortices, implicated in perseverance and reward. Participants' *growth mindset* positively correlated with dorsal striatal structural connectivity (113). These findings pose significant implications in neurorehabilitation. Grit and growth mindset are essential to conditions with long-term recovery trajectories involving motor (re)learning, and identifying structural connections that subserve these constructs may spur the development of targeted therapeutic approaches.

## Discussion

This review serves as a point of integration for self-efficacy and motivation literature, stroke rehabilitation, and neuroimaging. There exists an extensive array of evidence in the literature highlighting the influence of self-efficacy and motivation on post-stroke rehabilitation outcomes, including individuals' engagement and adherence to rehabilitation. Limitations in current self-efficacy and motivation assessment methods, combined with recent application of neuroimaging-based biomarkers in stroke, prompted a review of the neuroimaging literature to identify potential neural substrates underlying motivation and self-efficacy. This particular objective distinguishes this review from others. Research findings utilizing fMRI, fNIRS, and EEG revealed several pertinent anatomical structures and regions–several of which were associated with learning. Identifying ways to enhance an individual's self-efficacy and motivation has the potential to advance post-stroke rehabilitation and promote lasting behavioral change (119). Ascertaining neural substrates subserving these personal factors may benefit this effort.

An increasing number of clinical research studies in stroke rehabilitation recognize the value of patient-centered measures, reporting interactions between self-efficacy, participation, capacity (120, 121). The consideration of personal factors such as self-efficacy and motivation may adjudicate the disconnect between functional improvement in rehabilitation settings and enhanced participation in the natural environment. Additionally, factors of self-efficacy and motivation also determine how an individual copes with difficult situations during rehabilitation. Greater self-efficacy and motivation positively affect an individual's ability to manage and overcome stressful conditions (18, 42). As stroke rehabilitation focuses on an individual's voluntary movement, an individual's self-beliefs and attitudes are fundamental in determining rehabilitation outcomes. Moreover, these biopsychological factors also influence long term behavior change (122) and return to work post-stroke (123). Lastly, there is growing evidence of time and dose-dependent effects of rehabilitation on functional improvement post-stroke (124–126). As discussed earlier, self-efficacy and motivation influence an individual's engagement in and adherence to rehabilitation, and these factors may therefore also impact the treatment dosage that an individual receives. Therapists and other medical professionals involved in the rehabilitation process should therefore implement strategies in their clinical practice to foster self-efficacy and motivation among their patients (127). Components of the Take Charge intervention (63) and elements from ASAP (Accelerated Skills Acquisition Program) entailing the celebration of patient effort and collaboration between therapist and patient provide additional direction (128). Such strategies are particularly imperative in the inpatient rehabilitation setting where boredom is a common sentiment reported in individuals post-stroke (129). A systematic review examining inpatient post-stroke rehabilitation experiences from 560 participants across 10 countries attributed feelings of boredom, frustration, and powerlessness, in part, to the physical environment (i.e., rehabilitation setting) (130). Individuals frequently desired more activities, stimulation, and practice opportunities both during and after their therapy sessions. Purposeful structuring of the rehabilitation environment based on the individual may also cultivate motivation and self-efficacy during this critical period of recovery in addition to the patient-therapist relationship.

In order to leverage self-efficacy and motivation to advance stroke rehabilitation, there must first be accurate tools to assess these factors. Current assessment tools involve self-reported questionnaires wherein individuals must either response in yes or no or rate themselves on a Likert Scale. Considering the subjective nature of scoring and lack of standardization of scoring ranges in these assessments, these tools may have limited reliability and validity. Furthermore, such measures might have reduced accuracy in assessing self-efficacy and motivation due to possible response bias (131). This bias may be due to participant hesitancy or the need to give socially desirable answers. To mitigate these issues, utilizing neuroimaging-based measurements in conjunction with self-reported scores may provide more accurate characterization of self-efficacy and motivation post-stroke. Evidence supporting the use of neurologic biomarkers in combination with behavioral measure (85) along with recommendations from the Stroke Recovery and Rehabilitation Roundtable (SRRR) to incorporate biomarker data into future stroke recovery research (27) encourage the expansion of biomarker development to self-efficacy and motivation assessment.

In addition to potentially enhancing the objectivity of self-efficacy and motivation assessment, neural correlates of self-efficacy and motivation may also foster a more person-centered approach in motor learning and rehabilitation. Our review of the neuroimaging literature resulted in the identification of several potentially important structures and regions that require further investigation in individuals with stroke as the literature predominantly involved young adults without significant neurological history. Future work should confirm if similar brain-behavior associations reside in individuals with stroke. Further, similar to how biomarkers may differentiate treatment “responders” from “non-responders,” neural correlates of self-efficacy and motivation may also define those with varying levels of self-efficacy and motivation. Such information may inform an individual's treatment and recovery trajectory and also guide therapists in their delivery of feedback and structuring of tasks during therapy sessions. Neural structures and connections associated with self-efficacy and motivation may also guide clinical researchers in their development of stroke recovery prediction models and interventions. Given the relevance of self-efficacy and motivation in motor learning/control and stroke rehabilitation, inclusion of these personal factors in stroke recovery prediction models may enhance predictive performance and provide additional therapeutic targets.

The majority of studies reviewed utilized MRI and fMRI technologies. While these neuroimaging techniques have been widely utilized in stroke, their lack of accessibility and portability limit their application in a stroke rehabilitation setting. Technologies such as fNIRS and EEG may prove valuable in the examination of self-efficacy and motivation in a rehabilitation setting, particularly at the bedside, while also enabling researchers to examine various neural networks and connectivity-based measurements. Given the complexity of self-efficacy and motivation, it is likely that neural correlates underlying these factors extend beyond an anatomical structure.

To promote consistency across clinical trial methodology and outcomes research in stroke, the SRRR proposed a universal battery of assessments for researchers (132). Though the current battery does not contain participation measurements or baseline measures of self-efficacy or motivation, several function- and activity-specific measurements with documented psychometric properties exist: ABC Scale (133), Falls Efficacy Scale (134), Walk-12 (135), Short Self-Efficacy for Exercise Scale (136), and the Confidence in Arm and Hand Movement Questionnaire (137). For the purposes of obtaining a baseline measurement of general self-efficacy and/or motivation specific to stroke rehabilitation, we recommend the Stroke Self-Efficacy Questionnaire (SSEQ) (138) and the Stroke Rehabilitation Motivation Scale (SRMS) (25). Briefly, the 13-item SSEQ assesses self-efficacy beliefs related to everyday tasks and self-management (e.g., bed mobility, ambulation, dressing, coping with frustrations of stroke, and exercise adherence) (138). The questionnaire possesses high internal consistency (Cronbach's alpha = 0.90) and criterion validity with the Falls Efficacy Scale (Spearman's *r* = 0.803, *p* < 0.001) (138). The 28-item SRMS, adapted from the Sports Motivation Scale (139), assesses internal and external motivation (25). Though the scale demonstrated good inter-rater reliability and internal consistency, it is important to note the small sample size (*n* = 18) involved in the initial testing (25). Despite previous acknowledgment regarding the limitations of current self-efficacy and motivation assessments, obtaining baseline measures of self-efficacy and/or motivation is important since these factors influence constructive behaviors, attitudes, and beliefs in post-stroke recovery. Baseline knowledge of one's self-efficacy and/or motivation may inform recovery potential and treatment and learning responses while also serving as a potential covariate in a clinical trial. Thus, assessment of self-efficacy and motivation using self-reported measures in conjunction with objective neuroimaging-based measures have implications in both stroke rehabilitation practice and research.

## Conclusion

Self-efficacy and motivation are important factors in stroke recovery and rehabilitation. The therapist-patient relationship and rehabilitation setting play significant roles in nurturing self-efficacy and motivation in patients. The use of neuroimaging to identify potential neural substrates of self-efficacy and motivation will enrich our understanding of stroke recovery and rehabilitation. There is a limited but growing body of literature concerning neural substrates of self-efficacy and motivation, and this work collectively inspires additional work in clinical populations, including stroke, to generate novel research questions, experimental paradigms, and treatment targets to optimize post-stroke recovery and rehabilitation outcomes.

## Author Contributions

JMC conceptualized the paper. RG, ACa, and ACo conducted literature reviews. RG wrote the paper. ACa, ACo, and JMC edited the paper. All authors have read and approved the submitted version.

## Funding

This work was funded by the National Institutes of Health: R00HD091375 (JMC).

## Conflict of Interest

The authors declare that the research was conducted in the absence of any commercial or financial relationships that could be construed as a potential conflict of interest.

## Publisher's Note

All claims expressed in this article are solely those of the authors and do not necessarily represent those of their affiliated organizations, or those of the publisher, the editors and the reviewers. Any product that may be evaluated in this article, or claim that may be made by its manufacturer, is not guaranteed or endorsed by the publisher.
